# Tendinosis-like changes in denervated rat Achilles tendon

**DOI:** 10.1186/s12891-018-2353-7

**Published:** 2018-11-30

**Authors:** Roine El-Habta, Jialin Chen, Jessica Pingel, Ludvig J. Backman

**Affiliations:** 1 Department of Integrative Medical Biology, Section for Anatomy, Umeå University, Johan Bures väg 12, 901 87 Umeå, Sweden; 2 Department of Neuroscience, University of Copenhagen, København, Denmark; 3 Department of Community Medicine and Rehabilitation, Physiotherapy, Umeå University, Umeå, Sweden

**Keywords:** Collagen, Denervation, Rat, Substance P, Tendinosis

## Abstract

**Background:**

Tendon disorders are common and lead to significant disability and pain. Our knowledge of the ‘tennis elbow’, the ‘jumpers knee’, and Achilles tendinosis has increased over the years, but changes in denervated tendons is yet to be described in detail. The aim of the present study was to investigate the morphological and biochemical changes in tendon tissue following two weeks of denervation using a unilateral sciatic nerve transection model in rat Achilles tendons.

**Methods:**

Tendons were compared with respect to cell number, nuclear roundness, and fiber structure. The non-denervated contralateral tendon served as a control. Also, the expression of neuromodulators such as substance P and its preferred receptor neurokinin-1 receptor, NK-1R, was evaluated using real-time qRT-PCR.

**Results:**

Our results showed that denervated tendons expressed morphological changes such as hypercellularity; disfigured cells; disorganization of the collagen network; increased production of type III collagen; and increased expression of NK-1R.

**Conclusion:**

Taken together these data provide new insights into the histopathology of denervated tendons showing that denervation causes somewhat similar changes in the Achilles tendon as does tendinosis in rats.

## Background

Tendons are vital for human locomotion because they transmit force from contracting skeletal muscle to the bone [1]. Tendon injuries include overuse injuries (tendinosis) [2] and acute injuries (partial tear or rupture) [3]. Surgical repair of torn tendons or muscle-tendon units are technically very demanding surgeries and the healing process is challenging with a high risk of continued reduced functionality [4, 5]. One major complication in these injuries is denervation due to an injured nerve [6]. When it comes to denervation of the skeletal muscle, it has been reviewed thoroughly how denervation affects the muscle and creates atrophy, cell death and fat infiltration and consequently muscle weakness [7, 8]. But when it comes to the effects of denervation on tendon tissue, little is known. This is of interest to clinicians who treat patients with peripheral nerve injuries, as a better understanding of denervation and its consequences could prevent the development of tendon pathology and improve the outcome of rehabilitation interventions for these patients.

Denervation is distinguished from immobilization (e.g. inability to move a leg because of a bone fracture) by the fact that in denervation there is a loss of nerve supply to the muscle, whereas in immobilization the nerves are intact. Immobilization studies have shown increases of collagen turnover after immobilization [9]. However, studies measuring the cross-sectional area of the Achilles tendon both after short term (2 weeks) and long term immobilization (6 weeks) did not observe changes in the cross-sectional area of the tendon, in contrast to the muscle, which already showed significant atrophy after 2 weeks [10, 11]. These studies indicate that tendon tissue is more resistant to immobilization than the skeletal muscle is [10, 11]. There is evidence suggesting that denervation causes more severe changes of the tendon than immobilization does. For instance, in adult rats unilateral denervation caused a significantly increased collagen turnover and a significant loss of collagen (5–14%) after 3 month of disuse [12]. Furthermore, chemical denervation using Botulinum toxin injections have been shown to reduce the peak passive properties of the muscle tendon unit significantly in mice [13]. However, it is not clear whether denervation causes any morphologically and biochemically changes in denervated healthy tendons.

Nerve supply in the tendon is important for proper tendon function and is also related to the intrinsic healing capacity of tendons, specifically the sensory innervation in relation to tendinosis and the repair of ruptured tendons [14]. Normal and pathological tendons includes, in varying degree, sympathetic (adrenalin), parasympathetic (acetylcholine), and sensory (substance P) nerve fibers, as well as free nerve endings [15]. It is known that there is an extensive sprouting of nerve fibers into the healing tendon, which after completed healing regress from the tendon for the neuromodulators to return to baseline [16]. It is also shown that a healing tendon, completely lacking nerve supply, in the state of denervation, insufficiently heal and that the failure load of the tendon is decreased to 50% [17].

In the present study, we hypothesized that denervation would change both the morphological and biochemical features of the tendon significantly. In order to investigate our hypothesis we examined rat Achilles tendons two weeks following unilateral sciatic nerve transection. The cell number, nuclear roundness, and fiber structure were analyzed and compared with the contralateral healthy leg. Furthermore, the expression of different neuromodulators, including substance P and NK-1R, were analyzed.

## Methods

### Animals

Tendon biopsies were collected from six (*n* = 6) 10- to 12-week old female Sprague Dawley rats (Taconic Europe A/S) two weeks after unilateral sciatic nerve transection. The contralateral tendon served as a control. All surgical procedures were performed under isoflurane anesthesia (Attane vet, 1000 mg/g, Oiramal Healthcare, UK). Postoperative animals were given the analgesic Finadyne (Schering-Plough, Denmark, 2.5 mg/kg, s.c.) to minimize suffering. Animals were housed alone with free access to water and food, and were allowed to move freely so that the denervated limb could be loaded. As a consequence of denervation, animals loaded their entire foot instead of only the fore foot. Animals were euthanized by first inducing deep anesthesia using 4% isoflurane inhalation. When no response to stimuli was detected, the chest cavity was opened to induce pneumothorax in combination with cutting of the aorta to induce exsanguation. The rats were kept under isoflurane anesthesia until heartbeats had not been detected for five minutes. The animal care and experimental procedures were carried out in accordance with the Directive 2010/63/EU of the European Parliament and of the Council on the protection of animals used for scientific purposes. The study was also approved by the Northern Swedish Committee for Ethics in Animal Experiments (No. A186–12).

### Sampling and sectioning

After two weeks of denervation, the Achilles tendon was separated from the gastrocnemius muscle using a sharp razor blade. The cut was made 1–2 mm from the musculotendinous junction and the calcaneus to ensure no muscle or bone tissue was collected. Each tendon was cut longitudinally into two pieces, one for sectioning and one for real-time qRT-PCR. The tendon piece set aside for sectioning was mounted on thin cardboard in OCT embedding medium (Miles Laboratories) and frozen in propane chilled liquid nitrogen, and then stored at − 80 °C until further use. Sectioning was performed at − 22 °C using a cryostat-microtome. Series of 7 μm thick sections were collected and mounted on glass slides. The sections were processed for either immunohistochemistry or histological evaluation.

### Ex vivo analyses

Sections stained with hematoxylin-eosin were examined in a light microscope. Three different parameters were studied in the denervated tendon as well as the contralateral tendon: cell number, nuclear roundness, and fiber structure. Each section was given a score from 0 to 3, where 0 indicated no histological changes compared to normal tendon tissue, and 3 indicated changes commonly seen in tendinosis. This points-based system was adopted from Shen et al. [18] and was used to pinpoint the similarities and differences between the denervated and contralateral tendon.

### Cell number

For cell counting, photographs of hematoxylin-eosin sections were taken at 20x magnification and transferred to a computer for further processing. A grid was placed over each sample and three separate squares were counted, each with a size of 0.01 mm^2^. A mean value was then calculated, and sections of the denervated and contralateral tendon were compared to each other.

### Nuclear roundness

Using ImageJ (version 1.51, National Institutes of Health, USA) the nuclear length and width was measured, and a ratio of the two was calculated to determine the nuclear roundness. The cells were chosen randomly. In total, 100 cells were measured in each group before a mean value was determined.

### Fiber structure

To visualize the collagenous connective tissue fiber arrangement in tendon sections a trichrome stain was performed. Slides were placed in preheated Bouin’s Fluid for 60 min at 60 °C in a fume hood, followed by a 10 min cooling period. Slides were then rinsed in tap water until clear, plus once in distilled water, and stained with working Weigert’s Iron Hematoxylin for 5 min. After rinsing the slides in running tap water they were stained with the following solutions for 10 min each, with washing steps in-between: Biebrich Scarlet/Acid Fuchsin Solution; Phosphomolybdic/Phosphotungstic Acid Solution; and Aniline Blue Solution. Lastly, Acetic Acid Solution (1%) was applied to slides for 3 min, and then dehydrated very quickly in 2 changes of 95% Alcohol, followed by 2 changes of Absolute Alcohol. Slides were mounted in Pertex (Histolab Products AB, code: 00840). Nuclei was stained in black, organized collagen was shown in red, and disorganized collagen in blue, as previously described [19]. All sections were masked to blind the investigators before the histological evaluation.

### Immunohistochemistry

Sections for immunohistochemistry were fixed in 2% PFA, washed three times in PBS, and blocked in swine normal serum (1:20) for 15 min. Sections were then incubated with rabbit primary antibodies (type I collagen, 1:80, Abcam, ab34710; type III collagen, 1:100; Abcam, ab7778; and NK-1R, 1:50, Santa Cruz, SC-15323) for 60 min at 37 °C. Washing and blocking was repeated before the sections were incubated with TRITC-conjugated swine-anti-rabbit secondary antibodies (1:40, Dako, R0156) for 30 min at 37 °C. After a final washing step the slides were mounted in medium containing DAPI (Vector Laboratories, code: H-1200). Slides were examined in a Zeiss Axioskop 2 plus microscope equipped with an Olympus DP70 camera.

### Real-time qRT-PCR

A piece of the Achilles tendon was homogenized in QIAzol Lysis Reagent (Qiagen, code: 79306). The homogenization procedure was carried out using a handheld tissue ruptor for a couple of minutes until most of the tissue had dissolved. The homogenate was then placed at the benchtop for 5 min to promote dissociation of nucleoprotein complexes. Then, chloroform (1:5) was added to the tube and shaken vigorously for approximately 15 s. The homogenate was centrifuged at 18600 x *g* at 4 °C for 15 min, after which the upper aqueous phase was transferred to a new tube. After the addition of 1.5 volumes of 100% ethanol, total RNA was purified using RNeasy Mini Kit (Qiagen, code: 74106) according to manufacturer’s instructions. Reverse transcription was performed using TaqMan Reverse Transcription Reagents (Applied Biosystems, code: 4368813) from 1 μg RNA. Real-time qRT-PCR was performed with TaqMan Gene Expression Assay (Applied Biosystems) and ViiA 7 Real-Time PCR System (Applied Biosystems). Probes used included: *Collagen I* (Rn01463848), *Collagen III* (Rn01437681), *Tac1* (Rn01500392), and *Tacr1* (Rn00562004). Thermal-cycling conditions were 50 °C 2 min; 95 °C 20 s; and 40 cycles of 95 °C 1 s, and 60 °C 20 s. Data was analyzed with ViiA 7 Software (Applied Biosystems). Expression of the glycolytic enzyme Glyceraldehyde 3-phosphate dehydrogenase (Gapdh) was used as an internal control.

### Statistical analysis

Analyses of ex vivo tissue were from six rats. Results are presented as mean ± SD. Statistical analysis was made with GraphPad Prism 7 using Student’s t-test for paired samples. * indicates *p* < 0.05, ** indicates *p* < 0.01, *** indicates *p* < 0.001. Values of p < 0.05 were considered statistically significant.

## Results

### Denervated tendons display morphological changes

**Fig. 1:**
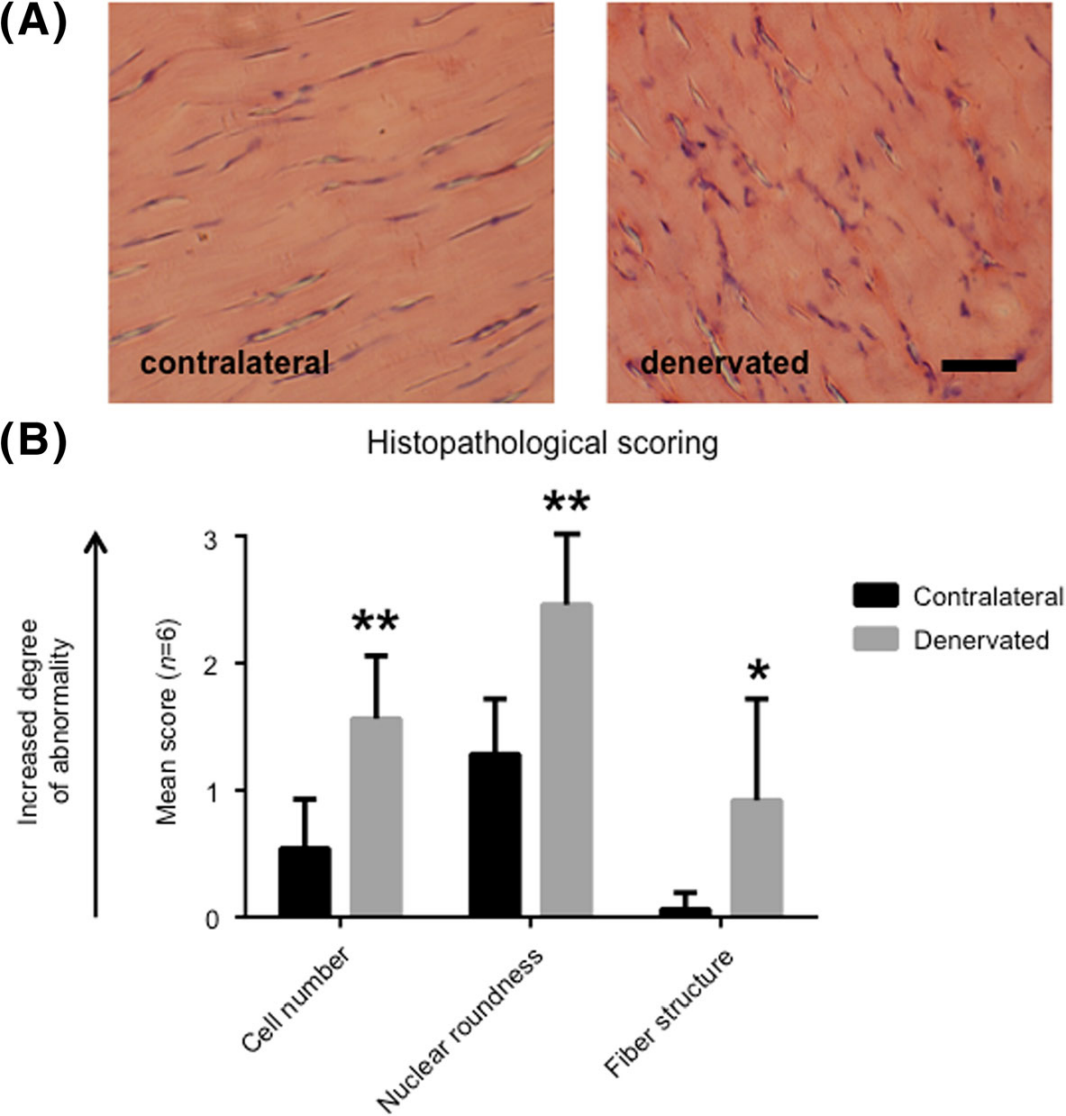
Histology of denervated rat Achilles tendon. (**a**) Hematoxylin-eosin staining of the middle part of the Achilles tendon two weeks after unilateral sciatic nerve transection. The rats were allowed to move freely post-surgery. Both sides, i.e. denervated and contralateral, are shown. Bar = 20 μm. (**b**) Histological sections were examined in a light microscope and given a score from 0 to 3 (0 = normal, and 3 = pathological). All examined parameters were significantly increased in the denervated group compared to the contralateral healthy leg. * indicates p < 0.05, ** indicates p < 0.01. Values of p < 0.05 were considered statistically significant

Frozen sections were prepared and stained with hematoxylin-eosin. As exemplified in Fig. 1a there were clear differences between the two groups, such as an increased number of cells, changes in nuclear roundness, and changes in collagen fiber structure. Each of these parameters was examined in more detail using a histological scoring system ranging from 0 to 3, i.e. from normal to pathological (Fig. 1b). These results confirmed that there are significant, tendinosis-like changes in denervated rat Achilles tendons two weeks following sciatic nerve transection.

### Significant increase in the number of cells in denervated tendons

**Fig. 2:**
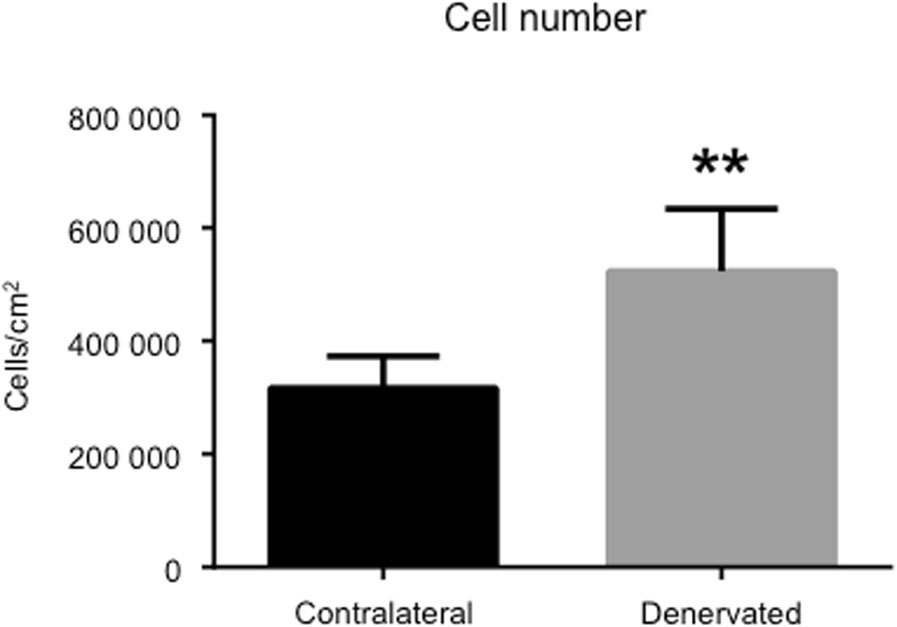
Pronounced increase in the number of cells in denervated tendons. Mean cell number from six independent animals. Denervated side compared to the contralateral side. Overall, there was a significant increase (approximately 70%) in the number of cells in denervated tendons compared to contralateral tendons. ** indicates p < 0.01. Values of p < 0.05 were considered statistically significant

For quantitative purposes, photographs of hematoxylin-eosin sections, as well as DAPI stains, were taken and the nuclei were counted in a computer where the cells could be marked to avoid double counting. Three representative areas from each section were chosen and a mean value was calculated. Data showed a significant increase in the number of cells in the denervated tendon compared to the contralateral tendon (Fig. 2).

### Increased nuclear roundness a common feature of denervated tendons

**Fig. 3:**
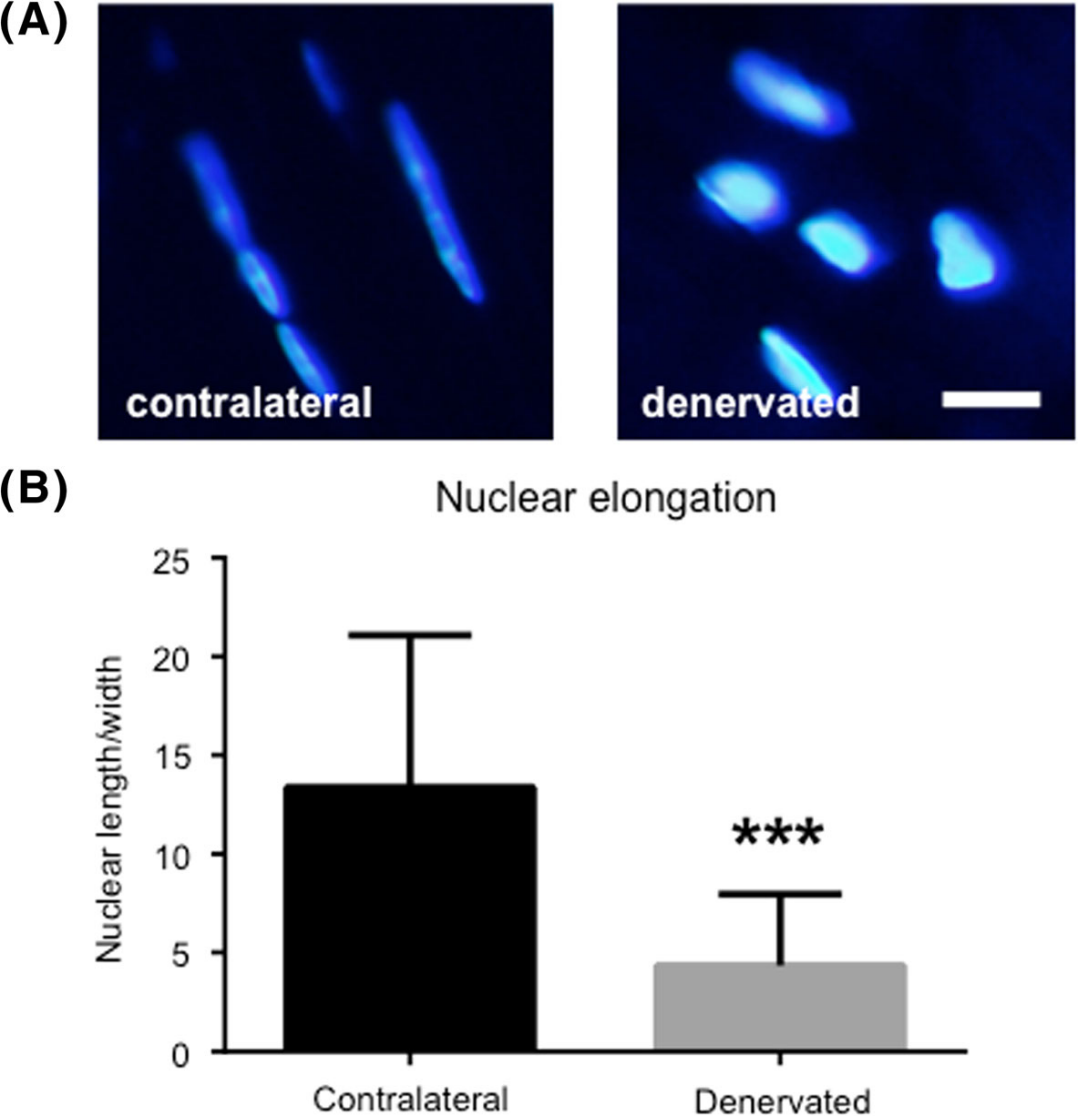
Increased nuclear roundness in denervated tendons. (**a**) Nuclei stained with DAPI revealing increased nuclear roundness in denervated samples compared to contralateral samples. Bar = 5 μm. (**b**) Ratio between nuclear length and width, measured with ImageJ. The lower the value, the greater the nuclear roundness is. *** indicates p < 0.001. Values of p < 0.05 were considered statistically significant

One prominent feature of denervated tendon was the nuclear roundness, which could be seen in sections from all six rats (Fig. 3a). Using ImageJ the length and width of each nuclei was measured. Results showed that cells in the denervated tendon had significantly lost their spindle shape and were more round than cells in the contralateral tendon (Fig. 3b).

### Disturbed collagen production and organization in denervated tendons

**Fig. 4:**
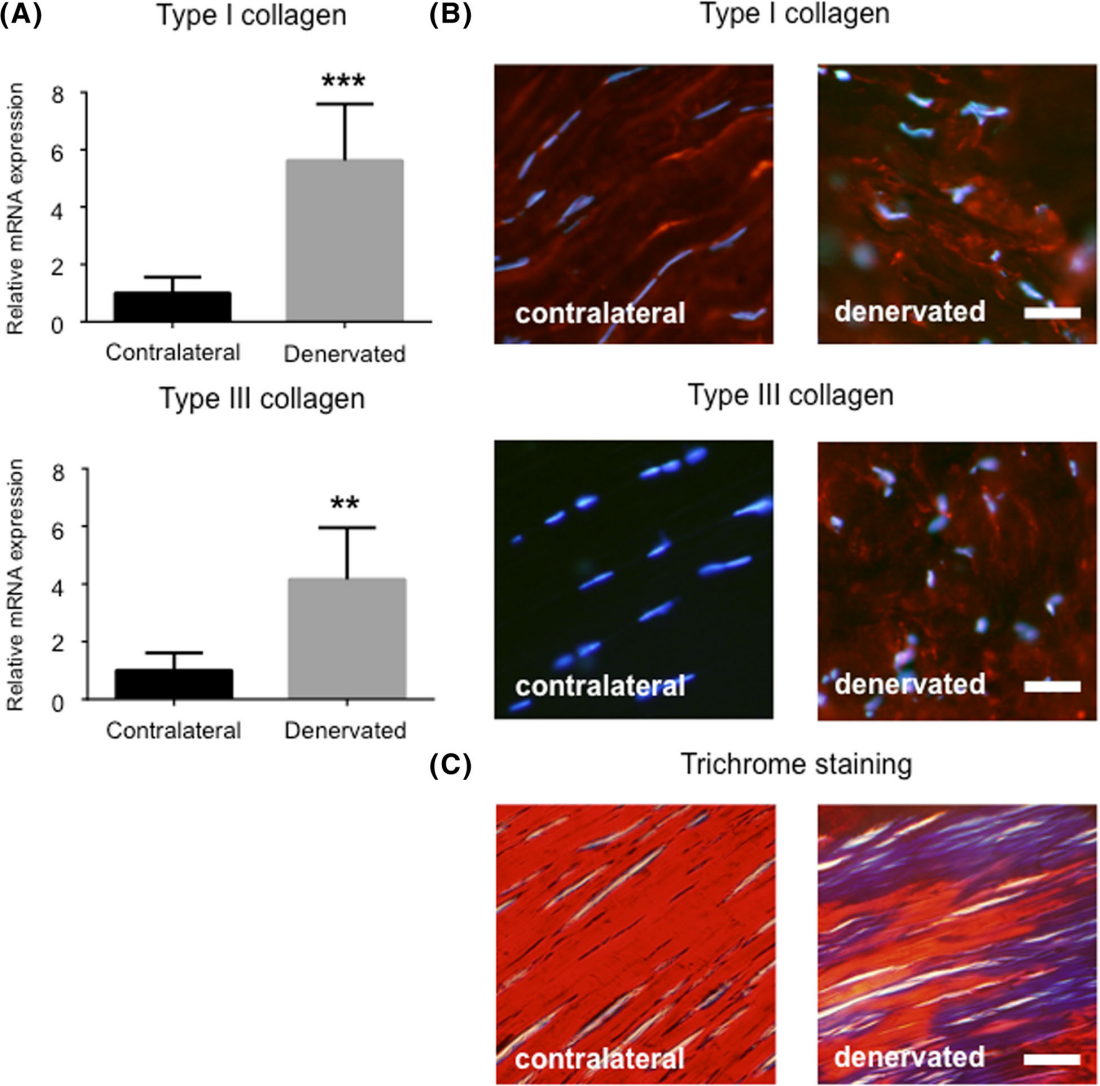
Disturbed production and organization of collagen in denervated tendons. (**a**) Increased mRNA expression of type I and type III collagen in denervated tendons. (**b**) Expression of collagen I and III protein (stained red) in contralateral (left column) and denervated (right column) tendons. DAPI was used to stain the nuclei (blue). Bar = 20 μm. (**c**) Trichrome stain revealing disorganized collagen (blue) in denervated tendons. Bar = 20 μm. ** indicates p < 0.01, *** indicates p < 0.001. Values of p < 0.05 were considered statistically significant

To evaluate the expression of type I and type III collagen, which is often altered in conditions such as tendinosis, qRT-PCR was performed on tendon biopsies from denervated rats. As shown in Fig. 4a*,* the expression of both types of collagen was up regulated in denervated samples compared to control samples. The production of collagen was then examined using immunohistochemistry, which confirmed the increased expression on the protein level (Fig. 4b). Additionally, trichrome stain was performed to visualize the collagenous connective tissue fibers. As can be seen in Fig. 4c, the dense and parallel structure of the contralateral tendon (red) is missing in the denervated tendon, which has more of a disorganized collagen structure (blue).

### Denervated tendons express the neurokinin 1 receptor

**Fig. 5:**
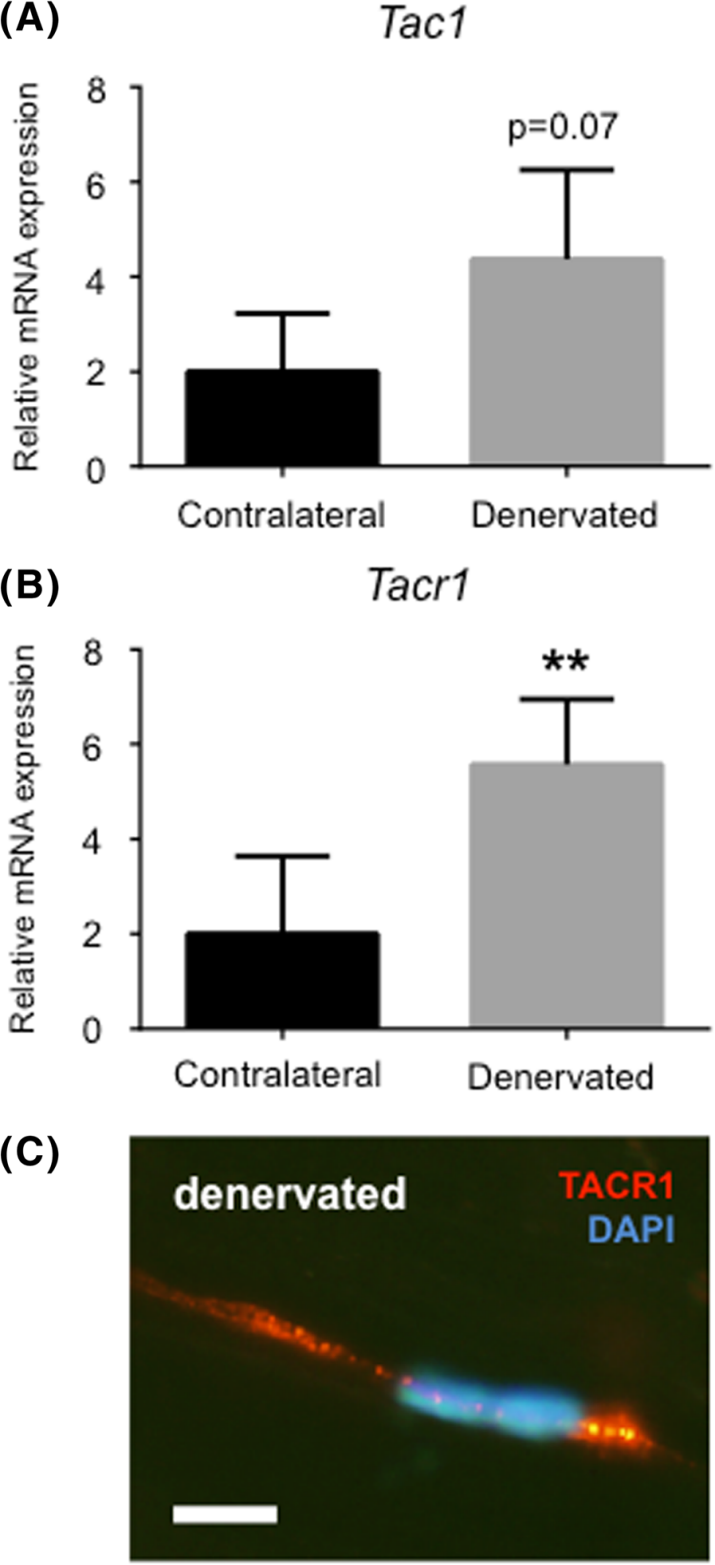
Denervated tendons express molecules associated with tendinosis. (**a**) Expression of genes related to tendinosis using qRT-PCR: substance P (*Tac1*) and its preferred receptor NK-1R (*Tacr1*). (**b**) Immunohistochemistry showing positive staining for NK-1R in a denervated tendon. Bar = 5 μm. ** indicates p < 0.01. Values of p < 0.05 were considered statistically significant

To further investigate the characteristics of denervated tendons and its possible connection to tendinosis, we performed qRT-PCR using markers related to tendinosis such as substance P and its preferred receptor Neurokinin-1. Results showed that denervated tendons expressed significantly more NK-1R (*Tacr1*) than contralateral tendons (Fig. 5a). NK-1R was also detected on the protein level in denervated samples, but not in contralateral samples (Fig. 5b). Substance P (*Tac1*) mRNA expression was also elevated, although not significantly (*p* = 0.07).

## Discussion

Here we present data regarding the histopathology of denervated rat Achilles tendons. For all morphological measures, including cell number, nuclear roundness, and fiber structure, denervated tendons received a significantly higher score than the corresponding contralateral tendon. Additionally, denervated tendons had a significantly higher expression of type I and type III collagen, and NK-1R. These findings indicate that rat Achilles tendons acquired tendinosis-like changes after two weeks of denervation. This is in line with previous observations of increased collagen turnover in tendons after denervation [9, 12]. In spinal cord injury patients a significant loss of dense connective tissue has been observed 1 year post injury [20] indicating that the increased turnover mainly might be collagen degradation. One important question is whether these changes are a result of neuromuscular changes or are due to immobilization alone. This can be very difficult to disentangle. One previous study observed that 9 weeks of immobilization caused an increased collagen turnover and further the tendon stiffness decreased significantly after immobilization, also indicating a significant loss of dense connective tissue [9].

In skeletal muscle tissue, denervation and immobilization cause significantly different changes [21]. In a study by Ceylan et al. it was observed that loss of muscle volume, weight, and length was significantly lower after denervation than after immobilization [21]. However, these differences were not seen when the Achilles tendons from the same chicken legs were analyzed after both treatments, only the length of the tendon was significantly lower in the denervated group compared to the immobilized group. This lack of significant difference might be partly explained by the time point chosen for this analysis. Since the tendon tissue changes its properties slower than skeletal muscle, 3 weeks might be too soon to measure significant differences between denervation and immobilization, especially with such rough parameters as volume, weight and length. In the current study we therefore focused on the morphological and biochemical changes of denervated tendons that often precede the macroscopical changes. It should also be noted that in our experiments rats were allowed to move freely post surgery so that the denervated limb could be loaded. Studies have shown that injury of the rat sciatic nerve does not completely compromise locomotion in this animal model [22]. However, because of denervation, the animals change the way they load both of their feet. As a consequence, one could speculate that the contralateral leg was loaded more post-surgery than pre-surgery. This could potentially lead to changes related to overuse in the contralateral leg.

One of the most prominent features of denervated tendons that were observed in the present study was an increase in the total number of cells (approximately 70% in denervated samples). This finding might indicate that denervation causes an inflammatory response, and that the increase in cell number might be an inflammatory process that triggers proliferation of tenocytes. However, these cells were not positively stained for CD68 (a macrophage marker) or mast cell tryptase. Exactly what kind of cells that infiltrates the tendon after denervation needs to be investigated further.

Another interesting finding was the difference in nuclear shape between denervated tenocytes and contralateral tenocytes. In denervated tendons the nuclei were generally less elongated, while they remained mostly unaffected in contralateral tendons. We suggest that this might be a consequence of disturbed collagen production and organization. Thus, we examined the expression and production of type I and III collagen using real-time qRT-PCR and immunohistochemistry and observed a significantly increased mRNA expression, as well as protein synthesis, in denervated samples. This was true for both types of collagen. Interestingly, there was no production of type III collagen in the contralateral tendon. The production of type III collagen is usually associated with tendinosis [23, 24]. Thus the present findings indicate that denervated tendons share some morphological features with tendons that suffer from tendinosis, as well as increased stiffness [25, 26]. From a clinical perspective this means that patients suffering from muscle atrophy due to neural damage would benefit from a rehabilitation intervention that takes the denervated tendon into account, i.e. treating the tendon as if it were a tendinosis tendon could improve the outcome of rehabilitation intervention and reduce the risk of developing tendon pathology. Type III collagen is also the first collagen type synthesized during the second stage of wound healing (proliferative phase) and is then replaced by type I collagen in the third and final stage (remodeling phase) [27]. The marked production of type I collagen in our denervated tendons could therefore be a sign of a transition from the second phase to the third phase of wound healing.

In the present study, the mRNA expression of NK-1R was significantly increased in denervated samples, and immunoreactions of NK-1R were observed. In a study by Bring et al. the expression of NK-1R was significantly increased in rats that had been mobilized after tendon rupture compared to the immobilized group [28]. The increase was approximately 3-fold in the mobilized healing group 17 days post-injury. This correlates well with our findings: a nearly 3-fold increase in the mRNA levels for NK-1R at 14 days post sciatic nerve transection. As for the expression of substance P, the mRNA was elevated (*p* = 0.07) but not to a significant level. It should however be mentioned that the variation of the substance P expression was very high between the subjects. While six rats created enough statistical power in order to measure significant differences in all morphological measures, the statistical power was too low to in order to measure statistical differences in substance P. However, it is still tempting to speculate on the importance of substance P in our case, especially since exogenously administered substance P is known to stimulate early reparative events, including cell proliferation [29]. Additionally, inflammatory cells have been shown to express substance P and NK-1R [30], which underlines our hypothesis that the increase in cell number might be explained by infiltration of inflammatory cells.

## Conclusions

To conclude, there are similarities between denervated tendons and tendinosis: an increased number of cells; cells that have lost their slender spindle-shape and look disfigured; disorganized collagen architecture; an increased production of type III collagen; and an increased expression of NK-1R. As discussed above, we do not know whether the observed alterations are due to neuromuscular changes, neurotropic interactions, or immobilization alone. Nevertheless, these changes should be taken into account in order to maximize the outcome for those suffering from peripheral nerve injuries and its complications. Clinically, methods to preserve the tendon morphology and function during denervation are important for successful rehabilitation once the nerve has re-innervated the target organ.

## Abbreviations

NK-1R: Neurokinin-1 receptor
qRT-PCR: Quantitative real-time polymerase chain reaction
SP: Substance P
